# FEELING YOUNG TODAY, FEELING GOOD TOMORROW? MICROLONGITUDINAL DYNAMICS IN SUBJECTIVE AGE

**DOI:** 10.1093/geroni/igac059.644

**Published:** 2022-12-20

**Authors:** Fiona Rupprecht, Laura Schmidt, Monika Sieverding, Jana Nikitin, Hans-Werner Wahl

**Affiliations:** University of Vienna, Vienna, Wien, Austria; Heidelberg University, Heidelberg, Baden-Wurttemberg, Germany; Heidelberg University, Heidelberg, Baden-Wurttemberg, Germany; University of Vienna, Vienna, Wien, Austria; Universität Heidelberg, Heidelberg, Baden-Wurttemberg, Germany

## Abstract

Insights into the short-term dynamics and micro-longitudinal consequences of subjective age can drive our understanding of its long-term mechanisms across adulthood. Using data from 80 newly retired individuals (aged 59 to 76 years; 59% women) collected on 21 days, we made use of a recent methodological advance—multilevel dynamic structural equation modeling. As possible same-day correlates and micro-longitudinal consequences of subjective age, we investigated physical activity, step number, sleep quality, affect, and stress, which were either assessed via wearables (FitBit Charge HR) or daily diaries. Analyses suggest a weak autoregression of subjective age, indicating that how old one feels is determined via daily rather than lasting experiences. Indeed, there were significant same-day relations to all suggested correlates. The one effect lasting across several days was from an older subjective age on subsequent negative affect—a potential short-term mechanism contributing to the detrimental long-term influence of an older subjective age.

